# Clinical Expectations for Better Influenza Virus Vaccines—Perspectives from the Young Investigators’ Point of View

**DOI:** 10.3390/vaccines6020032

**Published:** 2018-05-26

**Authors:** Kristin G.-I. Mohn, Fan Zhou

**Affiliations:** 1 Influenza Centre, Department of Clinical Science, University of Bergen, Bergen 5021, Norway; fan.zhou@uib.no; 2 Emergency Care clinic, Haukeland University Hospital, Bergen 5021, Norway; 3 K.G. Jebsen Centre for Influenza Vaccine Research, Department of Clinical Science, University of Bergen, Bergen 5021, Norway

**Keywords:** influenza, human, vaccines, immune response, future, clinical, safety

## Abstract

The influenza virus is one of a few viruses that is capable of rendering an otherwise healthy person acutly bedridden for several days. This impressive knock-out effect, without prodromal symptoms, challenges our immune system. The influenza virus undergoes continuous mutations, escaping our pre-existing immunity and causing epidemics, and its segmented genome is subject to reassortment, resulting in novel viruses with pandemic potential. The personal and socieoeconomic burden from influenza is high. Vaccination is the most cost-effective countermeasure, with several vaccines that are available. The current limitations in vaccine effectivness, combined with the need for yearly updating of vaccine strains, is a driving force for research into developing new and improved influenza vaccines. The lack of public concern about influenza severity, and misleading information concerning vaccine safety contribute to low vaccination coverage even in high-risk groups. The success of future influeza vaccines will depend on an increased public awarness of the disease, and hence, the need for vaccination—aided through improved rapid diagnositics. The vaccines must be safe and broadly acting, with new, measurable correlates of protection and robust post-marketing safety studies, to improve the confidence in influenza vaccines.

## 1. Influenza—Introduction

This year we commemorate the 100th anniversary of the devastating 1918 Spanish influenza pandemic, which is considered to be the deadliest single disease outbreak in humans of all times [1]. However, records of the respiratory illness that is caused by influenza viruses go back several hundred years. The early influenza epidemics were described as we do today, by their respiratory and systemic symptoms, as well as the typical acute onset and severe prostration, which help to differentiate this virus from the countless other respiratory viruses. Another clinical difference is the lack of prodromal respiratory symptoms, such as a congested or runny nose, which typically do not precede influenza. Many people without prior influenza infection wrongly self-diagnose a common cold as influenza, contributing to the underestimatioan of the burden of the disease. Globally, 5–10% of adults and 20–30% of children are infected by influenza every year, resulting in up to 650,000 deaths [2]. The fatality rates and the personal and socioeconomic costs of the annual influenza epidemics pose a considerable burden on society, with an estimated annual cost of 34.7 to 166.5 billion US $ in the United States (US) [3,4,5].

Influenza vaccines have been available for more than seven decades, and despite their shortcomings, are the most cost-effective countermeasure to mitigate transmission, illness, and fatal outcome of influenza. However, despite decades of research, current vaccines have limitations in effectivness and availability, which are the two most important factors for success. In addition, vaccine confidence and overall vaccination coverage is low even in high-risk groups [6,7]. The clinical demand for improved influenza vaccines is paramount. In this review, we will discuss the clinical expectations and challenges facing the future, improved influenza vaccines, and look at some of the promising next generation vaccines.

## 2. Vaccination Strategies and Currently Licenced Vaccines

Extensive research into host risk factors and viral virulence factors has been conducted to elucidate mechanisms causing severe human influenza disease. Studies have found a consistently increased risk of severe disease in pregnant women, people with chronic illnesses, obesity, and the elderly (>65 years old), and those with occupational risk [8]. In the US, Canada, and a few European countries, the influenza vaccine is recommended to all people >6 months and has been implemented into the childhood vaccination campaigns [9,10]. Most other European countries only vaccinate the high-risk population, and many low and middle-income countries do not have official vaccination policies [11]. 

Much is known about the influenza virus, and the two most important viral antigens so far, HA and NA, which are targets for vaccines. The influenza virus is an enveloped virus with eight segments of single-stranded RNA, which lacks proof-reading capability when the viral RNA genome undergoes replication in the host-cell nucleus. Thus, new virus progeny and their antigens undergo continuous mutations amid immune pressure, leading to “antigenic drift”, neccesitating biannual vaccine updating. Traditional, especially egg-based, vaccine production, and distribution takes several months. The choice of vaccine strains are made on predictions based on epidemiology surveillance and evolution modelling. Predictions of the dominant circulating strains are however imperfect, especially in case of the late emergence of antigenic drifted viruses, leaving populations vulnerable to mismatch between vaccine and circulating strains, as was seen for the H3N2 strain in 2014–2015 [2,12,13,14], and for the B strain in the 2017–2018 season in Europe [15]. Furthermore, “antigenic shift”, which is the reassortment of gene segments from different viruses, gives rise to novel viruses with pandemic potential due to the lack of immunity within the global population. New production methods based on cell cultures and viral vectors are being explored in order to increase production speed and quantity, as well as ease the burden of egg-dependent vaccine production [16,17].

**Figure 1:**
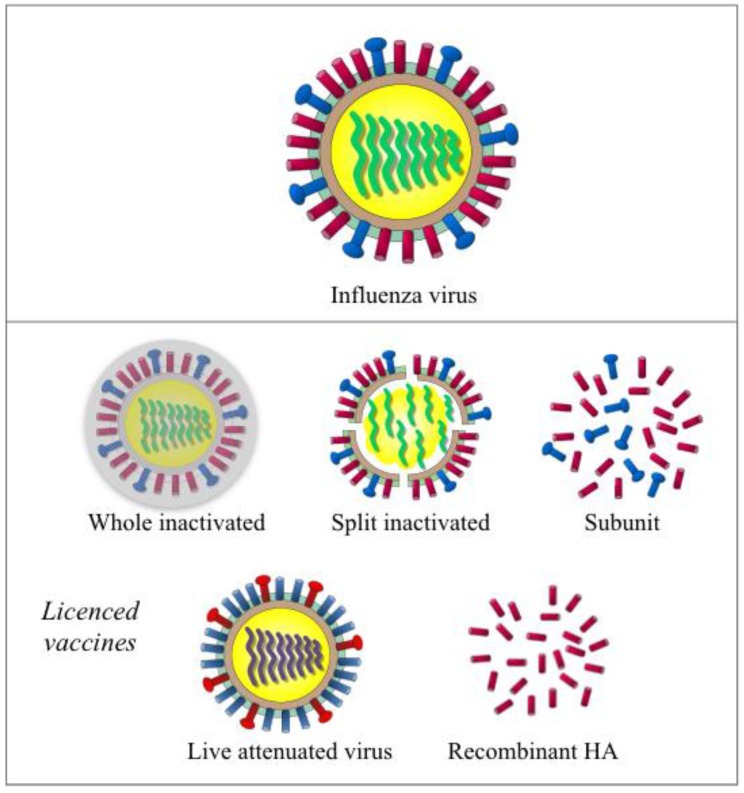
Illustration of the influenza virus and currently licensed vaccines. Influenza virus (the top panel) is a RNA virus with eight segments of negative sense single strand RNA genome. Two major surface glycoproteins hemagglutinin (HA, in red) and neuraminidase (NA, in blue) are the main antigenic components in inactivated influenza vaccines (the middle panel including whole inactivated, split inactivated and subunit vaccine). Live attenuated vaccine (left in lower panel) uses HA and NA that were expressed with the backbone of cold-adapted, temperature-sensitive attenuated master donor virus to elicit a multifaceted immune response. Recombinant-HA vaccine (right, lower panel) is produced in insect cells using a baculovirus vector.

Current influenza vaccines come in three types (inactivated, live attenuated, and recombinant-HA) and are designated to different populations (Figure 1). More than 140–170 million doses have been distributed annually in the US during the last five years [8]. 

An inactivated influenza vaccine (IIV) was initially developed in the 1940s and contained crudely purified, inactivated whole virus. Modern IIVs include whole, split, and subunit formulations, of these split and subunit IIVS are more commonly used due to improved tolerability. Split vaccines contain viruses, which are first inactivated with formalin or beta-propiolactone, are subsequently split with detergents. Subunit vaccines are further purified, to remove the lipids and nucleocapsids [18,19]. Egg-based IIVs have been licensed in the US and Europe for use in adults and children six months and older. MF59 adjuvanted or high dose IIVs are licensed for use in the elderly [20,21]. Cell-based IIVs are licensed in the US and Hungary for individuals that are allergic to eggs. IIVs mainly consists of two surface glycoproteins hemagglutinin (HA) and neuraminidase (NA), with varying amounts of nucleoprotein (NP) depending on different vaccine manufacturers [22]. 

Live attenuated influenza vaccine (LAIV) is licensed in the US and Canada (for use in adults and children two years and older), Europe (for use in children and adolescents, 2–17 years of age), Russia, and India [23]. In contrast to IIVs, LAIV is produced by reverse genetics, with six genome segments from the attenuated, temperature-sensitive cold-adapted master donor viruses (type A and type B), and HA and NA from the season’s circulating strains [24]. Multiple studies have proven LAIV to be genetically stable [25]. After intranasal administration, LAIV virions proliferate in the upper respiratory tract mimicking natural infection. In 2013, a recombinant-HA vaccine (FluBlok) was licensed for use in adults aged 18 to 49 years old in the US [26,27]. This baculovirus vector based vaccine is an improvement, being egg-free and having a shorter manufacturing timeframe when compared to IIV and LAIV.

## 3. To Mimic the Natural Immune Response to Influenza—The Goal of Future Vaccines? 

**Figure 2:**
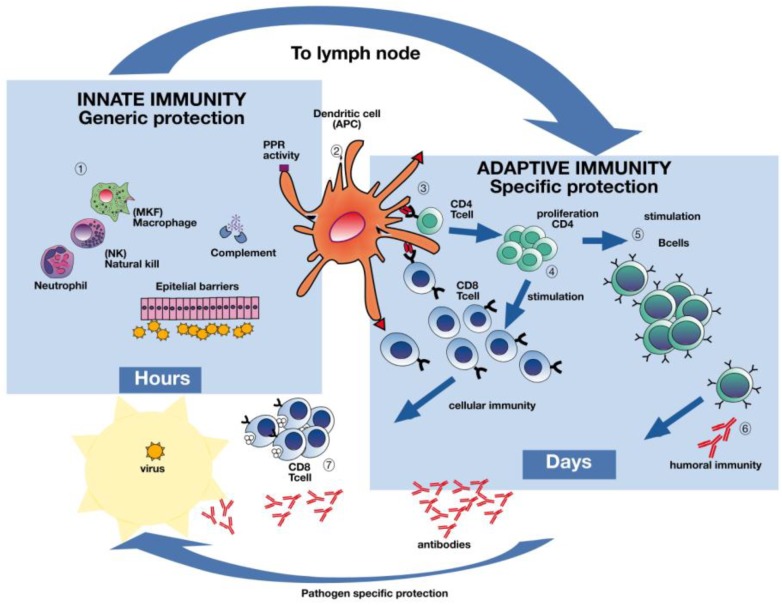
The immune response to influenza infection. Influenza virus infection elicits a multifaceted immune response. Live attenuated influenza vaccines activate both humoral and cellular immune responses while inactivated influenza vaccines predominantly elicit antibodies. Deeper understanding of the natural protective immune responses after influenza infection will aid in designing future influenza vaccines capable of activating both humoral and cellular immune responses covering several or possibly all influenza strains. Dendritic cells in the airway mucosa present influenza proteins both through the major histocompatibility complex classes I and II to CD4 and CD8 T-cells (Section 3). Antigen presenting cells (APCs) (Section 2) recognize the virus and the subsequent activation of pro-inflammatory cytokines occurs, inducing viral resistance in uninfected neighboring cells, as well as recruiting other immune cells (Section 1). The adaptive immune system will produce virus specific antibodies, as well as CD4 and CD8 T-cells, which are capable of destroying virus infected cells (Sections 4, 6, 7). Although slower, the adaptive immune system has memory and the capacity to recognize an unlimited number of antigens with great specificity, as opposed to the generic response of the innate immune system. Figure made in collaboration with GC Johnsen at the University of Bergen, Norway.

A vital factor determining the outcome of an influenza infection is the host´s initial adeptness to detect and restrict viral replication and the spread of progeny virus at the site of infection. The natural protective immune response to influenza is multifaceted and involves both strain specific antibodies and cellular immune responses to combat infection and symptom development (Figure 2). In order to design better vaccines, it is essential to gain better understanding of these complex immune responses that are induced by natural infection. 

Today´s IIVs have focused on achieving neutralizing antibodies (Figure 2 Section 6), targeted towards the receptor binding site of the globular head of HA, thereby inhibiting binding and entry of the virus into the cell [28]. Influenza strain specific antibodies protect from infection, and they have been the primary focus of influenza researchers for decades [29,30]. Indeed, the only agreed correlate of protection after influenza infection or vaccination is the Hemagglutination inhibition test (HI) and Microneutralization (MN) assays [31].

Two main problems with IIVs are their strain-specificity and variable vaccine effectiveness (VE). Meta-analyses have found that VE after IIVs vary from 60% in children and 40% in adults to matched strains [32]. This variability causes great public health concern and it contributes to the low vaccination uptake rate in general. The difference in effectiveness is due to several issues. Most importantly is the challenge with possible mismatch of vaccine- and circulating strain. A consequence of vaccine mismatch is the reduced vaccine efficacy, as was seen in the 2014–15 season, resulting in excess mortality in several countries [33,34]. Production of quadrivalent vaccines is an improvement, reducing the risk of B strain mismatch [35]. A mismatch in the B strain in trivalent vaccines was seen in Europe this season (2017/2018), where the vaccine strain contained the B Victoria lineage, while the dominating, circulating strain was the B Yamagata, resulting in excess hospitalizations in Norway and other European countries [15,36]. Populations with different HLA, as well as influenza immune history, pose other possible reasons for the low and variable VE of IIVs. 

LAIV has shown better immunogenicity than IIVs in children and only LAIV has been shown to induce both antibodies and T-cell responses in children (Figure 2 Sections 4–7) [37,38,39]. LAIV induces a broad and multifaceted immune response including antibodies in serum and the upper airway mucosa [40], as well as T-cells [41,42]. Cochrane reviews of LAIV have found ≈80% VE in children <6 years old and ≈40% in adults to matched strains [43,44]. LAIV mimics the natural immune response to influenza. Hence, LAIV has the potential to confer broader protection than the IIVs, especially in children [37,45]. Indeed, signs of herd immunity have been observed after LAIV childhood vaccination that commenced in the UK. It is believed that T-cell immune responses (Figure 2 Sections 3, 4, 7) are considered to confer this observed, protective effect [45,46]. It has been suggested that vaccination with LAIV could provide significant protection if faced with a new pandemic [47].

However, in the latter post-2009 pandemic years, LAIV VE in children has varied to such an effect that the Advisory Committee on Immunization Practices (ACIP) has gone from a preferential recommendation of the LAIV in 2014 to not recommending LAIV in 2016 [48,49], and is available for use next season after ACIP decision. The reason for the unexpected low VE towards H1N1pdm09 after LAIV is not clear, but the manufacturer updated the H1N1 strain that was used in LAIV vaccines from 2016, and future efficacy studies will indicate if this resolves the problem. Moreover, there is an unexplained disparity between LAIV vaccine effectiveness data from CDC and Europe. In Europe, LAIV has been found to provide lower protection than IIVs, however, moderate protection against H1N1pdm09 (41.5% in the UK and 47.9% in Finland) [50,51,52]. Similarly, a study from Senegal found that LAIV failed to protect against H1N1pdm09 in young children [53], whereas protection was found in a similar study in Bangladesh [54]. The reason for these differences could be related to the vaccine, the viruses, or the population´s exposure history [55,56]. The main difference between the US and Europe is the vaccine recommendations, with the US recommending influenza vaccination for everyone >6 months, while the European focus is on risk groups [57].

IgG is the most abundant circulating antibody isotype against influenza, and strain specific antibodies are measurable in the blood one-week post-vaccination or infection (Figure 2 Section 6) [58,59]. Secretory IgA is important in virus neutralization at the site of entry in the respiratory mucosa, and it is induced by LAIV administration [30,41]. Strain-specific antibodies inhibit infection, but have short effect due to the constant viral antigenic drift [60]. The problems with mismatch of vaccine and circulating strains, antigenic drift, and waning antibody response over time [61], and thereby the loss of protection from IIVs have led the global research field to search for broadly reactive antibodies to conserved regions of the HA and NA [62].

**Figure 3:**
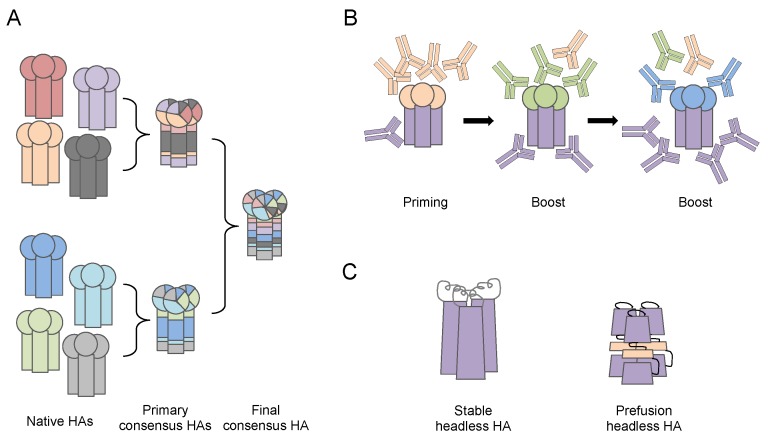
Illustration of antibody targeting next generation influenza vaccine designs. (**A**) Computationally optimized broadly reactive antigens (COBRA) strategy generates primary consensus hemagglutinin (HA) amino acid (aa) sequences from HAs of native circulating influenza strains, and further secondary and final consensus HA aa sequences from the primary consensus HAs based on a computational algorithm and antigenic evolving analysis. The de novo HA proteins with consensus aa sequence can be displayed on virus-like-particles or rescued influenza viruses using reverse genetics. (**B**) Chimeric HA. Conserved HA stalk domain from a circulating virus is combined with an irrelevant HA head domain from viruses that are absent in humans. The priming-boost-boost immunization regimen enhances immunogenicity of the subdominant HA stalk, thus elicits cross-reactive stalk specific antibodies. (**C**) Stable and pre-fusion Headless HA. Mini-HA proteins are engineered to have a correct-folded structure of the stable or pre-fusion stage of HA protein stalk domain. The immunogenicity of HA stalk is greatly enhanced by removing the HA head domain entirely.

Some of the next generation vaccine designs aim at more broadly reactive antibodies against the dominant head region of HA or the more conserved stalk region (Figure 3). Ross and colleagues have developed computationally optimized broadly reactive antigens (COBRA) by utilizing multiple rounds of a consensus generating algorithm to tackle the large antigenic variations in the HA head. The de novo COBRA HA proteins that were displayed on the surface of virus-like particles elicited broadly neutralizing antibodies, and provided protection in naïve and pre-exposed murine and ferret models [63,64,65]. Due to the minimum alteration in HA conformational structure, the implementation of this COBRA strategy in the currently approved IIV production pipeline has potential, making it a promising new candidate influenza vaccine. Others aim to achieve a truly universal vaccine by targeting the conserved stalk region of HA. Krammer and colleagues have used novel chimeric HA antigens containing the conserved stalk (from circulating H1N1 or H3N2 viruses) and irrelevant heads (from influenza viruses not infecting human) to overcome the low immunogenicity of HA stalk. The vaccine induced broadly neutralizing antibodies and provided protection in murine and ferret models [66]. Other research groups, using advanced protein engineering, established methods for making correctly folded stable or pre-fusion stage HA proteins containing only the stalk region, the so called “headless HA”. These headless HAs elicit stalk specific antibodies and they provide protection against the heterologous influenza virus challenge in murine and ferret models [67,68,69].

In the later years, the research focus on influenza immunity has widened from protective antibody responses to understanding the cellular response to influenza infection [70,71,72,73]. An important milestone on the way to develop a “universal” influenza vaccine will depend on developing a vaccine that is capable of inducing broad cross-reactive protection associated T-cell responses, which could mitigate the problem of vaccine strain specificity [74]. Both CD4 and CD8 T-cells have been linked to reduced severity of influenza disease (Figure 2 Sections 4 and 7), and different T-cell response patterns have been found in severely infected and mildly infected or vaccinated people [75]. The vaccinated and mildly infected patients showed similar immune response patterns, differing from patients with severe disease [75].

CD4 T-cells represent a key factor in limiting influenza disease due to their cytotoxic destruction of infected host cells and the stimulation of B-cells and CD8 T-cells (Figure 2 Section 7) [76,77]. CD8 T-cells cannot inhibit infection per se, however, they play an important role in limiting disease severity and the reduction of virus shedding by killing of virus infected host cells and clearing of the infection (Figure 2 Sections 3–7). CD8 T-cell subsets were linked to less severe pandemic infection in 2009 and to increased survival after H7N9 infection in China [70,78]. 

The phenomenon of heterosubtypic immunity [79,80,81], where host immune responses towards conserved viral epitopes cover several influenza subtypes, is of foremost interest in the development of next generation influenza vaccines. Although heterosubtypic antibodies have been shown to occur and to play a role in heterosubtypic immunity [82,83,84], historically cross-reactive cytotoxic T lymphocytes recognizing conserved internal epitopes have been the major focus of research [85,86]. Numerous studies demonstrate that individuals possess CD4 and CD8 T-cells with cross-reactivity to influenza A virus strains for which they have not been previously exposed [74,87,88,89,90,91]. 

Unsurprisingly, considerable research into next generation influenza vaccines has concentrated on harnessing cross-reactive T-cells in development of broadly protective vaccines. Recent studies have been targeting internal viral proteins, which are mostly conserved in structure and amino acid sequences, but are less immunogenic. Different platforms, such as virus-like particles [92], adenoviral vectors (Ad5 and ChAdOx 1), and modified vaccinia vector (MVA) have been utilized to enhance the cellular immune responses [93,94,95]. One leading candidate here is the MVA-NP+M1, in which NP and matrix protein 1 (M1) are expressed using the replication-deficient modified vaccinia virus Ankara (MVA) vector. Previous studies have proven good safety and tolerance of the MVA vector [96], and the MVA-NP+M1 has been able to elicit potent NP and M1 specific cellular immune responses [94]. A recent study demonstrated that the co-administration of seasonal influenza vaccine and MVA-NP+M1 simultaneously, achieved potent antibody and T-cell responses in adults that were aged 50 years and older [97]. On the other hand, Xavier Saelens and colleagues set the goal of eliciting cross reactive humoral and cellular immune response by targeting NA and extracellular domain of matrix protein 2 (M2e) at the same time. The M2 protein is highly conserved, making it an especially interesting vaccine target. Virus-like particles carrying NA and M2e have been reported to elicit both serum IgG and CD8 T-cells and provide protection against heterologous virus challenge in a murine model, but the effect in humans remains to be elucidated [92].

Albeit difficult to conduct, future research on the immune responses in naturally infected patients will help to decipher the complex immune response to influenza, and aid in directing an optimized future vaccine response. Such research will also aid in finding new correlates of protection for evaluation of future vaccines [76,98].

## 4. Adjuvants

Due to the purity of the antigens in current vaccines, they have excellent safety profiles, however their immunogenicity can be low. Adjuvants ameliorate the uptake and presentation of weak antigens, improving the vaccine induced immune responses [99,100]. These factors are important in achieving a sufficient immune response in the elderly, immunocompromised, and the young [101,102]. Adjuvanted vaccines increase the breath of the antibody response and help overcome pre-existing antibodies [103]. The adjuvants that are licensed for use in human influenza vaccines are aluminum, oil-in-water, and squalene based formulations MF59, AS03, and AFO3 [104,105,106]. For influenza vaccines, the oil-in-water emulsion adjuvants (MF59 and AS03) have proven to be more effective in increasing immune responses and hence dose sparing [105], and both were used in the monovalent pandemic vaccines in 2009. MF59 adjuvanted IIV has been recently licensed in the US for use in the elderly and showed better immunogenicity than non-adjuvanted [106]. Current adjuvants target the innate immune system. The development of new adjuvants, which could enable robust humoral and cellular immune responses, is considered to be important for future vaccine development. 

## 5. Safety 

Currently licensed vaccines are well-tolerated and are considered safe, with mostly transient, local side effects, such as stuffy or runny nose (for LAIV) or soreness and redness at the injection site (for IIVs) [107,108,109]. Vaccines are given prophylactically to healthy people and to children; hence, the tolerance for side effects is very low. Historically, safety concerns have influenced vaccine uptake, and with rapid information spread through the mass media, there is increased public questioning about safety issues concerning vaccines in Europe, with decreasing vaccine coverage, highlighted by a recent Lancet editorial [110]. The same has been observed in Australia, where parents’ confidence in influenza vaccines was lower in 2010–2012 than in 2008–2009 due to a manufacturer reporting adverse events in young children in 2010 [111,112]. In order to inform the public and to uphold confidence in the annual influenza vaccination campaigns, increased attention must be given to lessen the knowledge gap between researchers and policy makers. In addition, surveillance systems of adverse events that are linked to vaccination are of vital importance. The association between Pandemrix (AS03-adjuvanted 2009 pandemic influenza vaccine) and increased incidences of narcolepsy in children and adolescents in Europe was discovered thanks to robust post-market surveillance systems [113,114,115,116,117,118]. The Pandemrix vaccine was not licensed in the US and narcolepsy was not linked to the US licensed non-adjuvanted pandemic vaccines [119]. In Europe, the cost and the accessibility of influenza vaccines is not considered to be a major hurdle for vaccination. However, the confidence in vaccines is [110]. 

## 6. Challenge: Lack of Knowledge due to Clinical Underdiagnosis of Influenza

Seasonal influenza vaccination is voluntary and self-financed in most countries and the uptake is far below the WHO recommended level of 75% coverage [120]. A major hurdle for increased influenza vaccine acceptance rate is the varying VE rates and that influenza is not perceived as a major threat to the public. In Europe, influenza has until recently been under diagnosed, with patients that are admitted to hospital most often diagnosed with “viral illness”, camouflaging influenza among other respiratory viruses. Importantly, the development of high throughput, sensitive PCR tests, has improved accuracy and reduced the time to diagnosis in patients admitted to hospital with influenza, adding to increased public awareness of severe disease [121]. Improved diagnostics along with information campaigns about the risk to personal and public health that could aid in the public education is needed. 

## 7. Our Perspective for the Expectations for Future Influenza Vaccines

The much needed, improved future influenza vaccines, can be divided into different developmental groups, depending on the public health goal. If the goal is a “one shot fix all” universal influenza vaccine that covers all influenza strains, a novel method of vaccine engineering is needed. Such a vaccine would have a neutralizing effect, thus inhibiting infection. However, if invented, it could be difficult to sell to the ever updated and side-effect orientated public.

In light of the European experience with the AS03-adjuvanted pandemic vaccine, we believe that implementing a potential novel, universal influenza vaccine in Europe will be challenging, as the general vaccination coverage in Europe is in decline [110]. Although tempting from the researchers or public health point of view, with a potential great impact on combating influenza, we fear that the public will be hesitant. With a novel vaccine comes the risk of unknown and rare serious adverse events that must not be underestimated. The induction of broad antibody responses against conserved influenza components, as well as new elements in delivery vectors, increases the risk of cross-reactivity with self-proteins, and hence severe adverse events [122]. Therefore, extra care should be taken in pre-clinical and clinical safety studies.

Another vaccination goal to consider is whether protection from severe disease (hospitalization) and fatal illness would be equally rewarding but less complicated, and then to attempt to achieve sterilizing immunity with a novel vaccine. Such a strategy could entail focusing on a combination of improving the LAIV vaccine for children, and targeting different IIV formulations for the appropriate risk grups. With novel vaccines requiring several years before licensing, incremental steps with a focus on individual vaccination recommendations could be beneficial. Instead of a “one shot fix all” approach, targeted usage of improved “old” vaccines would perhaps be more acceptable for the public and would provide better protection. If future influenza vaccines could be improved to elicit both broadly cross-reactive antibodies as well as T-cell responses, we would be a step closer to a universal vaccine. Perhaps a combination of a quadrivalent LAIV (QLAIV) and a the most effective IIV could achieve this goal? 

The LAIV has shown to elicit multifaceted humoral and cellular immune responses in influenza naïve populations without pre-existing mucosal IgA blocking viral replication and subsequent immune responses [41,123]. Therefore, LAIV could be suggested for children as a priming vaccine [124]. Moreover, the LAIV is likely to offer indirect protection in the elderly and reduce transmission of the virus in the community. Such signs of herd immunity have been observed in the UK after LAIV implementation in school-age children [10,125]. IIVs, on the other hand, do not require replication, activate pre-existing memory B- and T-cells eliciting more potent antibody responses than LAIV. Therefore, IIVs are more suited as a boost regime for people with influenza exposure history through infection or vaccination, and could be recommended for older children/young adults. The recently licensed quadrivalent inactivated influenza vaccine (QIV) is an additional improvement, removing the challenge of choosing B lineage, thus reducing influenza B infections and the impression of the low vaccine effect in the public. This may boost vaccination confidence and uptake. QIVs could be provided to adult risk groups and pregnant women, while the elderly could receive the MF59 adjuvanted or high-dose IIV, which has shown better protection in this risk group.

Such improvements combined with personalized vaccination regimes in parallel to increasing vaccination coverage would reduce the personal burden of influenza illness, as well as the burden on health structures. Improving current standard of care vaccines is a safe and achievable approach to improve influenza protection in the general public.

## 8. Conclusions

As we are about to commemorate the 100 the anniversary of the 1918 influenza pandemic, we continue to lack an effective vaccine. Future influenza vaccines aim to overcome the strict strain specificity of today’s vaccine and to provide cross-protective immune responses, protecting against several strains and lasting more than one influenza season. The success for clinical usage and acceptance by the public of new vaccines will depend foremost on vaccine safety and increased awareness of the severity of influenza infection. This will include improved, rapid, bedside diagnostic assays, and educating the public in order to motivate those at the highest risk of severe infection to be vaccinated. Deeper understanding of the complex immune response to natural infection will aid in reaching the goal of a universal influenza vaccine.
